# Incremental Value of Radiation Dose for Prognostic Stratification Beyond Performance Status in Palliative Radiation Therapy for Bone Metastases

**DOI:** 10.1016/j.adro.2026.102170

**Published:** 2026-10-04

**Authors:** Yui Watanabe, Takuya Tomoda, Akiko Iwata, Hirokazu Matsuno, Hiroto Hayakawa, Takeshi Nagata

**Affiliations:** Department of Radiology, Daiyukai General Hospital, Ichinomiya, Aichi, Japan

## Abstract

**Purpose:**

Although the individual prognostic value of performance status (PS) and prescribed dose for overall survival (OS) in palliative radiation therapy for bone metastases has been investigated, their combined prognostic significance has not been thoroughly explored. This study aimed to evaluate the combined prognostic value of PS and prescribed dose for OS in patients with bone metastases undergoing palliative radiation therapy.

**Methods and Materials:**

This study included 423 patients who underwent palliative radiation therapy for bone metastases between January 2013 and October 2024. The primary endpoint was OS, evaluated at fixed follow-up points of 1, 3, and 6 months, as well as 1, 2, and 3 years.

**Results:**

Univariate Cox regression analysis demonstrated that 1-year OS was significantly associated with poor PS (hazard ratio, 2.6 [95% CI, 2.2-3.1]; *P* < .001) and a lower prescribed dose (<39 Gy in a biologically effective dose with α/β = 10; hazard ratio, 3.9 [95% CI, 2.8-5.3]; *P* < .001), both of which remained independent predictors of worse OS in multivariate analysis (both *P* < .001). Comparison of the areas under the curves showed that adding prescribed dose to PS significantly improved the predictive accuracy of OS at 1 and 3 months (both *P* < .001), 6 months (*P* = .002), 1 year (*P* = .028), and 2 years (*P* = .032). However, the improvement was not statistically significant at 3 years (*P* = .086). Kaplan–Meier curve analysis with log-rank tests showed that combining PS and prescribed dose improved OS risk stratification (*P* < .001 for all follow-up periods), regardless of primary tumor risk or the number of distant metastases.

**Conclusions:**

Combining PS and prescribed dose enabled more refined risk stratification for predicting short- and long-term OS in patients with bone metastases undergoing palliative radiation therapy, regardless of primary tumor risk classification or the number of distant metastases. Moreover, adding prescribed dose to PS provided significant incremental predictive value for OS in this setting.

## Introduction

Bone is a principal site for distant cancer metastases, third in incidence only to the lung and liver.^1^, ^2^, ^3^ Metastatic involvement predominantly affects the vertebrae, pelvis, ribs, and the metaphyseal regions of long bones, a distribution largely attributed to the high concentration of hematopoietic red marrow at these skeletal sites.^4^, ^5^, ^6^ In the spine, lesions most frequently occur in the lumbar region (52%), followed by the thoracic (36%) and cervical spine (12%).^7^ Radiation therapy (RT) is widely employed for the palliation of pain arising from bone metastases.

Patients undergoing palliative RT for bone metastases have a wide range of performance status (PS), which is influenced by the presence and number of distant metastases, as well as other clinical factors such as age and comorbidities. PS is one of the most widely used clinical indicators for guiding treatment decisions and estimating prognosis in oncology practice.^8^, ^9^, ^10^, ^11^, ^12^ Beyond its role in daily clinical assessments, PS is also incorporated into clinical trial eligibility criteria,^13^ reflecting its central importance in oncology research. Moreover, PS interacts with various patient-related factors, including age, nutritional status, and comorbidities, reinforcing its importance in prognostic stratification. Numerous studies have identified PS as a significant prognostic factor in patients with metastatic cancer undergoing palliative RT,^14^, ^15^, ^16^ consistently demonstrating that a favorable PS is associated with improved survival outcomes.

Palliative RT for bone metastases is delivered using various fractionation schedules, such as 30 Gy in 10 fractions, 20 Gy in 5 fractions, and 8 Gy in a single fraction. The choice of a specific regimen is often guided by factors such as patient prognosis, symptom severity, and institutional protocols, contributing to considerable heterogeneity in clinical practice. Several retrospective studies have reported an association between higher prescribed palliative radiation doses and longer survival.^17^, ^18^, ^19^ However, this association should be interpreted with caution, as patients with a more favorable expected prognosis may have been preferentially selected for longer-course or higher biologically effective dose (BED) regimens. Despite these findings and the potential for selection bias, the optimal fractionation strategy remains a subject of ongoing debate. Consequently, current international guidelines^20^ permit flexibility in regimen selection, emphasizing the need for further evidence to clarify the prognostic role of prescribed dose in palliative settings.

Although the prognostic significance of PS and the association between prescribed dose and overall survival (OS) have been investigated separately, as noted above, their combined prognostic significance in patients with bone metastases receiving palliative RT has not been fully elucidated. This study therefore aimed to evaluate the combined prognostic value of PS and prescribed dose for OS in this specific patient population.

## Methods and Materials

### Study design

This single-center, retrospective study reviewed patients who received palliative RT for bone metastases between January 2013 and October 2024. The study protocol was approved by the institutional review board (approval number: 2025-010), which granted a waiver of informed consent due to the retrospective nature of the study. All research procedures complied with applicable ethical standards. Clinical and dosimetric data were obtained from the institutional radiation oncology database.

A total of 484 patients met the initial eligibility criteria. Exclusion criteria were as follows: (1) receipt of stereotactic body RT for bone metastases (n = 1), and (2) patients lost to follow-up within 1 month after RT initiation (n = 60; patients who died within 1 month were included in the analysis). The final study population comprised 423 patients.

### Treatment

Computed tomography simulation was performed with patients in the supine position using an Aquilion LB scanner (Canon Medical Systems) with a 2 mm slice thickness. RT planning was performed on the Pinnacle treatment planning system (version 9.10; Philips Medical Systems). To facilitate comparison across fractionation schedules, the prescribed dose was converted to BED using an assumed α/β ratio of 10 (BED10). For patients who did not complete the intended RT course, the delivered dose was estimated by multiplying the planned dose by the proportion of fractions actually delivered. Fractionation schedules were selected at the discretion of the treating radiation oncologist according to routine institutional practice. Regimen selection was individualized based on the overall clinical context, including treatment intent, symptom burden, prior irradiation, feasibility of repeated visits, and other relevant patient- and treatment-related factors. No uniform prospective protocol or predefined institutional algorithm was used to assign patients to a specific dose level based on PS or predicted survival. In this study, a high prescribed dose was defined as BED10 ≥ 39 Gy, corresponding to 30 Gy in 10 fractions or higher. The most frequently used regimen was 30 Gy in 10 fractions. All treatments were delivered using 3-dimensional conformal RT with 4 to 10 MV photon beams generated by an Elekta Synergy linear accelerator.

Treatment volumes were defined as follows: gross tumor volume (GTV) included the visible tumor on planning computed tomography, with reference to available diagnostic imaging (magnetic resonance imaging or nuclear imaging). The clinical target volume (CTV) included the GTV and the relevant anatomical compartment containing potential microscopic disease. For vertebral metastases, the CTV typically included the entire involved vertebral body. For nonvertebral metastases, the CTV consisted of the GTV with an appropriate margin extending to the boundaries of the involved bone segment. The planning target volume was created by adding a 5 to 10 mm margin to the CTV to account for setup and motion uncertainties; for vertebral metastases, the planning target volume was often extended to include the adjacent superior and inferior vertebral bodies. Organs at risk, such as the spinal cord and bowel, were contoured, and dose constraints were applied in accordance with institutional protocols to minimize treatment-related toxicity.

### Statistical analysis

The follow-up period was calculated from the date of RT initiation. The primary endpoint was OS, defined as the interval from the start of treatment to death from any cause. Patients who remained alive were censored at fixed time points: 1, 3, and 6 months and 1, 2, and 3 years posttreatment. Based on previously published prognostic models for patients with bone metastases,^14^^,^^16^^,^^21^, ^22^, ^23^ primary tumors were classified into 2 prognostic risk groups according to expected survival: low-risk primary tumor types, including breast cancer, prostate cancer, and hematologic malignancies, and high-risk primary tumor types comprising all other tumor types. In this classification, “low-risk” and “high-risk” refer to primary tumor-related prognostic risk rather than radiosensitivity or radioresistance. Specifically, this classification was used because primary tumor type is a well-established prognostic factor in patients with bone metastases. Because lesion-level metastatic counts and longitudinal progression status were unavailable, true oligometastatic or oligoprogressive disease could not be rigorously identified. In a supplementary analysis, patients were stratified by the number of nonbone metastatic site categories (≤1 vs ≥2), and Kaplan–Meier analyses were performed for each subgroup.

Cox proportional hazards regression models were used to estimate hazard ratios and corresponding 95% CIs in both univariate and multivariate analyses. A *P* value ≤ .05 was considered indicative of statistical significance. Receiver operating characteristic curve analysis was performed to evaluate the predictive accuracy of PS alone and in combination with prescribed dose for OS, based on the area under the curve (AUC). Survival probabilities were estimated using the Kaplan–Meier method, and intergroup comparisons were made using the log-rank test. All statistical analyses were performed using R software (version 3.6.1; R Foundation for Statistical Computing).

## Results

### Patient characteristics

The demographic and clinical profiles of the 423 patients enrolled in this study are summarized in Table 1. Unless otherwise indicated, percentages reported in this paragraph were calculated using the full cohort of 423 patients as the denominator. The median age was 71 years (range, 36-93), with 223 patients (52.7%) aged ≥71 years. Men comprised the majority of the cohort, accounting for 260 patients (61.5%). Distribution of the Eastern Cooperative Oncology Group PS (ECOG PS) scale was as follows: PS 0 to 1: n = 152 (35.9%); PS 2: n = 104 (24.6%); and PS 3 to 4: n = 167 (39.5%). Lung cancer was the primary tumor site, found in 185 patients (43.7%), followed by breast (n = 63; 14.9%), prostate (n = 54; 12.8%), and colorectal (n = 33; 7.8%) cancers. Tumors categorized as low risk, including breast cancer, prostate cancer, and hematologic malignancies, were observed in 120 patients (28.4%), while the remaining 303 patients (71.6%) had high-risk tumors involving other primary sites. Most patients (n = 351; 83.0%) presented with multiple bone metastases. The number of distant metastatic sites excluding bone was distributed as follows: 0 sites: n = 171 (40.4%); 1 site: n = 119 (28.1%); and ≥2 sites: n = 133 (31.4%). A detailed distribution of nonbone metastatic site categories, ranging from 0 to 6, is shown in Table E1. Among nonbone metastatic sites, the lung was the most frequently involved organ (n = 116; 27.4%), followed by the liver (n = 112; 26.5%) and nonregional lymph nodes (n = 92; 21.7%). Vertebral bones were the most frequently irradiated site (n = 237; 56.0%), followed by the pelvis (n = 118; 27.9%), extremities (n = 43; 10.2%), ribs/sternum (n = 35; 8.3%), and skull (n = 12; 2.8%). Regarding prescribed radiation dose (BED10), 39 Gy was administered to 63.1% of patients (n = 267), <39 Gy to 16.3% (n = 69), and >39 Gy to 20.6% (n = 87).

**Table 1:** Patient characteristics

Characteristics	Patients, n (%) (n = 423)
Age (y)	
<71	200 (47.3)
≥71	223 (52.7)
Sex	
Men	260 (61.5)
Women	163 (38.5)
ECOG PS	
0-1	152 (35.9)
2	104 (24.6)
3-4	167 (39.5)
Primary tumor sites	
Lung	185 (43.7)
Breast	63 (14.9)
Prostate	54 (12.8)
Colorectal	33 (7.8)
Kidney	18 (4.3)
Esophagus/gastric	11 (2.6)
Oral/pharyngeal	10 (2.4)
Bladder	8 (1.9)
Liver (HCC)	9 (2.1)
Pancreatic/hepatobiliary	4 (0.9)
Ovary	3 (0.7)
Renal pelvis/ureter	11 (2.6)
Hematologic malignancy	3 (0.7)
Uterus	5 (1.2)
Pulmonary artery sarcoma	2 (0.5)
Pleura (malignant pleural mesothelioma)	1 (0.2)
Cancer of unknown primary	3 (0.7)
Risk of primary tumor	
High-risk group	303 (71.6)
Low-risk group	120 (28.4)
No. of bone metastases	
1	72 (17.0)
≥2	351 (83.0)
No. of distant metastases	
0	171 (40.4)
1	119 (28.1)
≥2	133 (31.4)
Site of distant metastases	
Brain	51 (12.1)
Lung	116 (27.4)
Liver	112 (26.5)
Adrenal gland	39 (9.2)
Nonregional lymph nodes	92 (21.7)
Pleural dissemination	54 (12.8)
Peritoneal dissemination	15 (3.5)
Others	87 (20.6)
Sites of palliative RT for bone metastases	
Vertebral	237 (56.0)
Pelvis	118 (27.9)
Extremity (eg, femur or humerus)	43 (10.2)
Ribs/sternum	35 (8.3)
Skull	12 (2.8)
Others	21 (5.0)
Prescribed dose (BED10)	
<39.0 Gy	69 (16.3)
8 Gy × 1 fraction	14 (3.3)
4-6 Gy × 3-6 fractions	31 (7.3)
2-2.5 Gy × 5-15 fractions	9 (2.1)
3 Gy × 1-9 fractions	15 (3.5)
39.0 Gy	267 (63.1)
>39.0 Gy	87 (20.6)
History of prior irradiation	
Any prior irradiation	15 (3.5)
By prescribed dose group (BED10)	
<39.0 Gy	7 (10.1)
39.0 Gy	6 (2.2)
>39.0 Gy	2 (2.3)

*Abbreviations:* BED10 = biologically effective dose with α/β = 10; ECOG PS = Eastern Cooperative Oncology Group Performance Status; HCC = hepatocellular carcinoma; RT = radiation therapy.

### Evaluation of symptom relief, adverse events, and functional status

Pain response at the irradiated site was evaluated as a local symptomatic outcome according to international consensus criteria defined in Tables E2 and E3. As shown in Table E4, overall pain response rates, defined as a complete or partial response, were 162 of 326 patients (49.7%) at 1 month, 116 of 239 patients (48.5%) at 3 months, and 92 of 188 (48.9%) at 6 months after RT. When comparing overall response with pain progression, no statistically significant differences were observed among the 3 prognostic groups at any time point. These findings suggest that the OS stratification achieved by combining ECOG PS and prescribed radiation dose was not accompanied by clear between-group differences in local pain response and pain progression. In terms of safety, the overall incidence of acute adverse events was low, with no grade 3 or higher toxicities recorded across the entire cohort (Tables E5 and E6). Additionally, functional preservation analysis showed that most patients maintained stable PS at 1 month posttreatment (Table E7). Finally, detailed characterization of poor PS subsets confirmed no significant imbalance in the proportion of patients with ECOG PS 3 to 4 between groups 2 and 3 (Table E8).

### Univariate and multivariable models for OS

Table 2 presents results from univariate and multivariate Cox proportional hazards regression analyses to identify factors associated with 1-year OS. During the follow-up period, 238 deaths were recorded among 371 patients. In the univariate analysis, several factors were associated with significantly poorer survival outcomes, including male sex, poor PS, high-risk primary tumor type, a greater number of distant metastases, the presence of liver or adrenal gland metastases, and a lower prescribed dose (<39 Gy in BED10) (all *P* < .001). Additionally, a higher number of bone metastases (*P* = .002) and the presence of brain (*P* = .008), lung (*P* = .001), or nonregional lymph node (*P* = .003) metastases were also significantly associated with poorer OS.

**Table 2:** Cox proportional regression analysis for 1-year overall survival

	Univariate		Multivariate	
Variables	*P* value	HR (95% CI)	*P* value	HR (95% CI)
Age ≥ 71 y	.050	1.3 (1.0-1.7)		
Men	<.001	1.7 (1.3-2.2)	.355	1.1 (0.9-1.5)
ECOG PS (0-1, 2, 3-4)	<.001	2.6 (2.2-3.1)	<.001	2.3 (2.0-2.8)
High-risk primary tumor type (vs low risk)	<.001	2.2 (1.6-3.0)	<.001	2.2 (1.5-3.1)
No. of bone metastases (≥2 vs 1)	.002	1.8 (1.2-2.7)	.001	2.0 (1.3-3.0)
No. of distant metastases (0, 1, and ≥2)	<.001	1.6 (1.3-1.8)	.063	0.7 (0.6-1.0)
Site of distant metastases				
Brain	.008	1.6 (1.1-2.3)	.100	0.7 (0.5-1.1)
Lung	.001	1.6 (1.2-2.1)	.040	1.5 (1.0-2.1)
Liver	<.001	2.6 (2.0-3.4)	<.001	2.4 (1.7-3.4)
Adrenal gland	<.001	2.1 (1.4-3.1)	.201	1.3 (0.9-2.1)
Nonregional lymph nodes	.003	1.6 (1.2-2.1)	<.001	2.1 (1.4-3.1)
Pleural dissemination	.156	1.3 (0.9-1.8)		
Peritoneal dissemination	.214	1.6 (0.8-3.2)		
Prescribed dose (BED10) (<39.0 Gy vs ≥39.0 Gy)	<.001	3.9 (2.8-5.3)	<.001	2.6 (1.9-3.6)

*Abbreviations:* BED10 = biologically effective dose with α/β = 10; ECOG PS = Eastern Cooperative Oncology Group performance status; HR, hazard ratio.

Among the variables that showed significant associations with poorer OS in the univariate Cox regression analysis, the following were identified as independent predictors of OS in the multivariate analysis: poor PS (*P* < .001), high-risk primary tumor type (*P* < .001), a greater number of bone metastases (*P* = .001), the presence of lung (*P* = .040) or liver (*P* < .001) metastases, nonregional lymph node metastases (*P* < .001), and a lower prescribed dose (<39 Gy in BED10) (*P* < .001). In contrast, male sex (*P* = .355), a higher number of distant metastases (*P* = .063), and the presence of brain (*P* = .100) or adrenal gland (*P* = .201) metastases were not independent predictors of OS.

Subgroup analyses of 1-year OS stratified by clinical factors such as age, sex, the number of distant metastases, and the presence of specific metastatic sites are illustrated in Fig. E1. Across all evaluated subgroups, a lower prescribed dose (<39 Gy in BED10) was consistently associated with a trend toward poorer survival.

### Comparison of AUCs for OS prediction

As shown in Table 3, comparison of AUCs demonstrated that adding the prescribed dose (BED10) to ECOG PS significantly improved the predictive accuracy of OS at most time points: 1 and 3 months (both *P* < .001), 6 months (*P* = .002), 1 year (*P* = .028), and 2 years (*P* = .032). In contrast, improvement was not statistically significant for 3-year OS (*P* = .086).

**Table 3:** Comparison of AUCs for predicting overall survival using ECOG PS alone versus ECOG PS plus prescribed BED10 at different time points

	ECOG PS alone	ECOG PS plus prescribed BED10	
Follow-up period	AUC (95% CI)	AUC (95% CI)	*P* value
1 mo	0.79 (0.75-0.83)	0.89 (0.85-0.91)	<.001
3 mo	0.80 (0.76-0.83)	0.85 (0.81-0.89)	<.001
6 mo	0.80 (0.76-0.84)	0.83 (0.79-0.86)	.002
1 y	0.80 (0.76-0.84)	0.82 (0.78-0.86)	.028
2 y	0.79 (0.75-0.84)	0.82 (0.77-0.86)	.032
3 y	0.73 (0.67-0.78)	0.75 (0.70-0.80)	.086

*Abbreviations:* AUC = area under the curve; BED10 = biologically effective dose with α/β = 10; ECOG PS = Eastern Cooperative Oncology Group Performance Status.

### Kaplan*–*Meier curve analysis combining PS and radiation dose

Figure 1 illustrates OS stratified by combinations of ECOG PS and prescribed radiation dose. Patients were classified into 3 groups based on the latter 2 factors: group 1 included those with a good PS (<2) and a high prescribed dose (≥39 Gy in BED10); group 2 comprised patients with either a poor PS (≥2) and a high prescribed dose or a good PS and a low prescribed dose (<39 Gy in BED10); and group 3 consisted of those with both a poor PS and a low prescribed dose. As shown in Fig. 1, Kaplan–Meier curves showed that patients in group 3 had significantly poorer OS than those in group 1. Patients in group 2 had intermediate survival outcomes compared with groups 1 and 3. These stratified Kaplan–Meier curves were evaluated at 1 month (Fig. 1A), 3 months (Fig. 1B), 6 months (Fig. 1C), 1 year (Fig. 1D), 2 years (Fig. 1E), and 3 years (Fig. 1F) of follow-up, with all comparisons showing statistically significant differences (*P* < .001).

**Figure 1:**
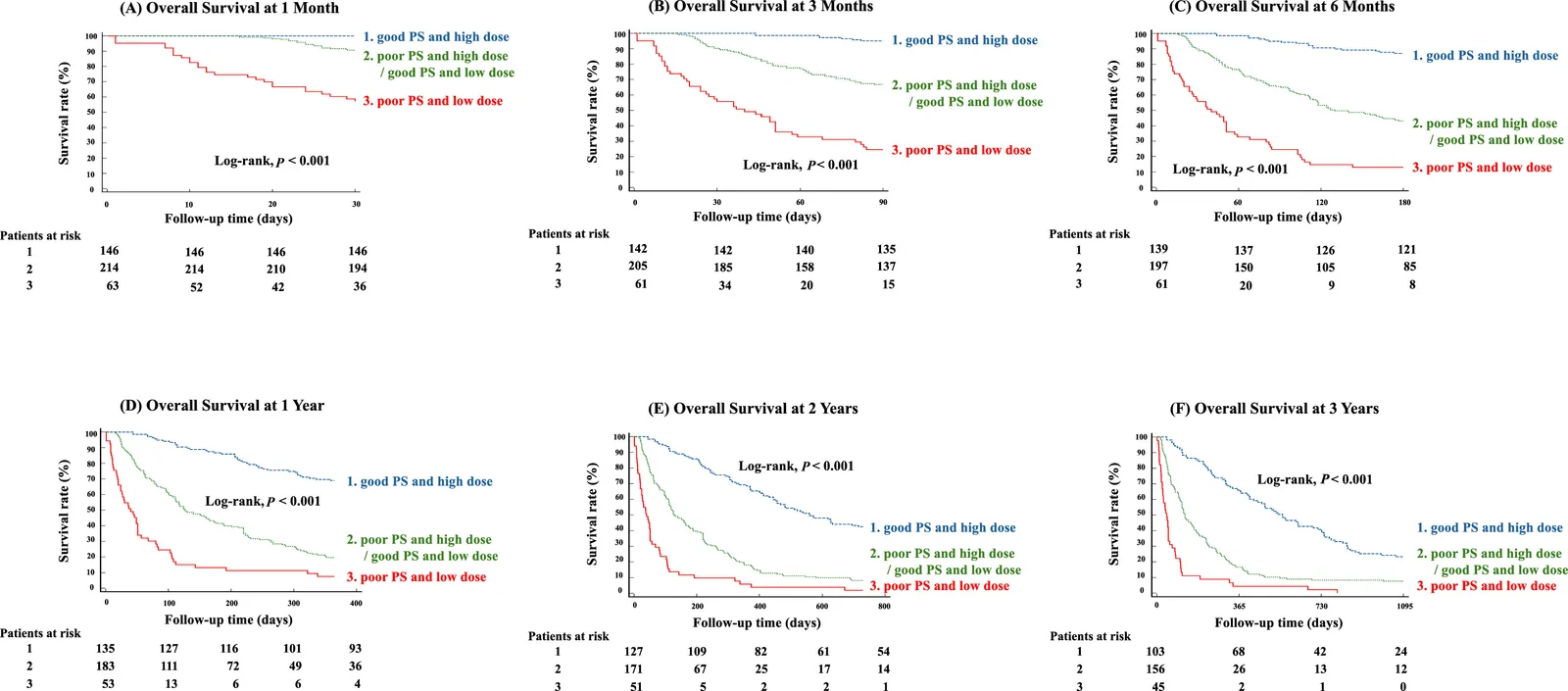
Kaplan–Meier curve analysis combining performance status (PS) and radiation dose in all patients. Kaplan–Meier curves showing overall survival in all patients, stratified by combinations of Eastern Cooperative Oncology Group Performance Status and prescribed radiation dose. Patients were categorized into 3 groups based on these 2 factors: group 1 included those with a good PS (<2) and a high prescribed dose (≥39 Gy in a biologically effective dose with α/β = 10); group 2 included patients with either a poor PS (≥2) and a high dose or a good PS and a low dose (<39 Gy in a biologically effective dose with α/β = 10); and group 3 included those with both a poor PS and a low dose. Survival was analyzed at (A) 1 month, (B) 3 months, (C) 6 months, (D) 1 year, (E) 2 years, and (F) 3 years posttreatment, with all comparisons showing statistically significant differences (log-rank *P* < .001).

Similarly, subgroup analyses were conducted over the same follow-up periods as in Fig. 1. Figure 2 shows patients with high-risk primary tumor types; Fig. 3, those with low-risk primary tumor types; Fig. 4, those with both bone and other-site metastases; and Fig. 5, those with bone-only metastases. All comparisons demonstrated statistically significant differences in OS among groups (*P* < .001). In the supplementary analysis stratified by nonbone metastatic site burden, a lower prescribed dose (<39 Gy in BED10) remained significantly associated with poorer 1-year OS in patients with ≤1 and ≥2 nonbone metastatic site categories (both log-rank *P* < .001; Fig. E2).

**Figure 2:**
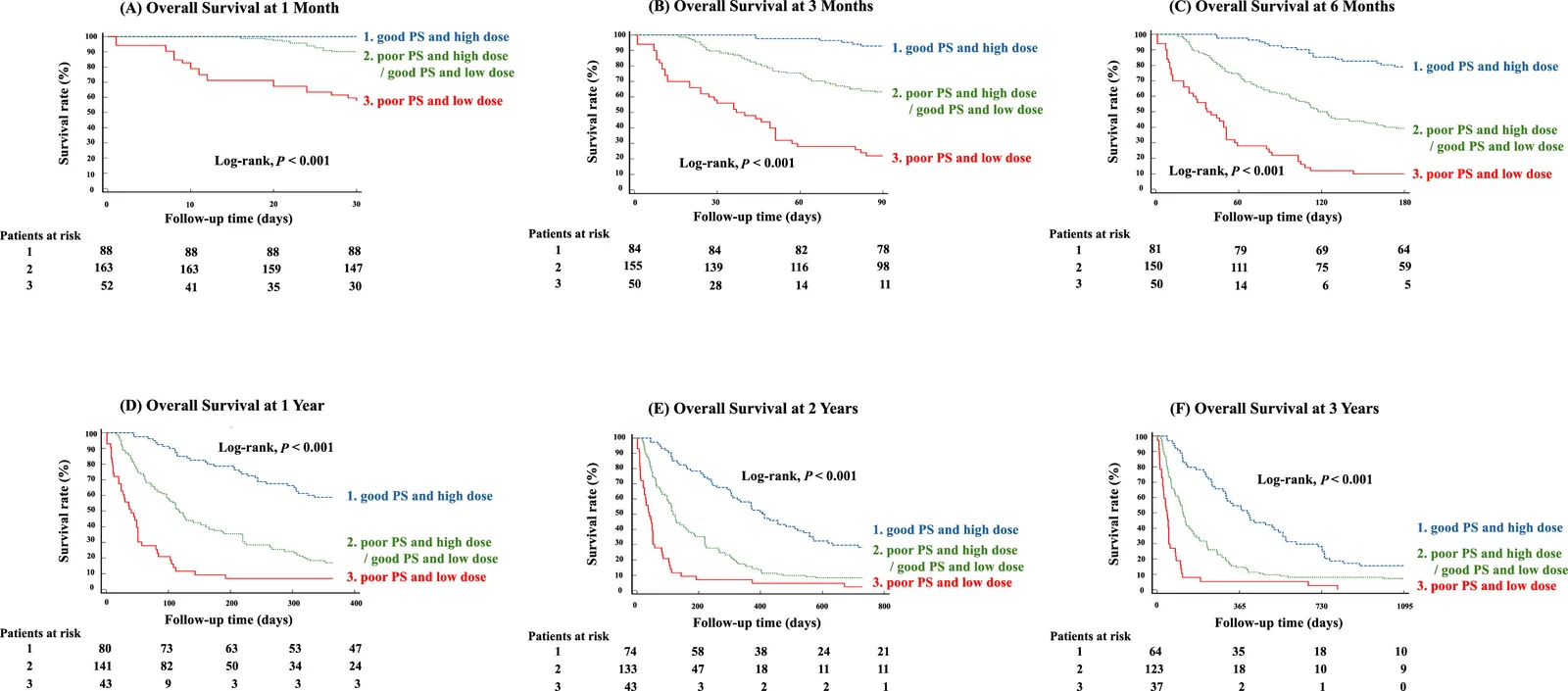
Kaplan–Meier curve analysis combining performance status (PS) and radiation dose in patients with high-risk primary tumor type. Kaplan–Meier curves showing overall survival in a subset of patients with high-risk primary tumor types, stratified by combinations of Eastern Cooperative Oncology Group Performance Status and prescribed radiation dose. Patients were categorized into 3 groups based on these 2 factors: group 1 included those with a good PS (<2) and a high prescribed dose (≥39 Gy in a biologically effective dose with α/β = 10); group 2 included patients with either a poor PS (≥2) and a high dose or a good PS and a low dose (<39 Gy in a biologically effective dose with α/β = 10); and group 3 included those with both a poor PS and a low dose. Survival was analyzed at (A) 1 month, (B) 3 months, (C) 6 months, (D) 1 year, (E) 2 years, and (F) 3 years posttreatment, with all comparisons showing statistically significant differences (log-rank *P* < .001).

**Figure 3:**
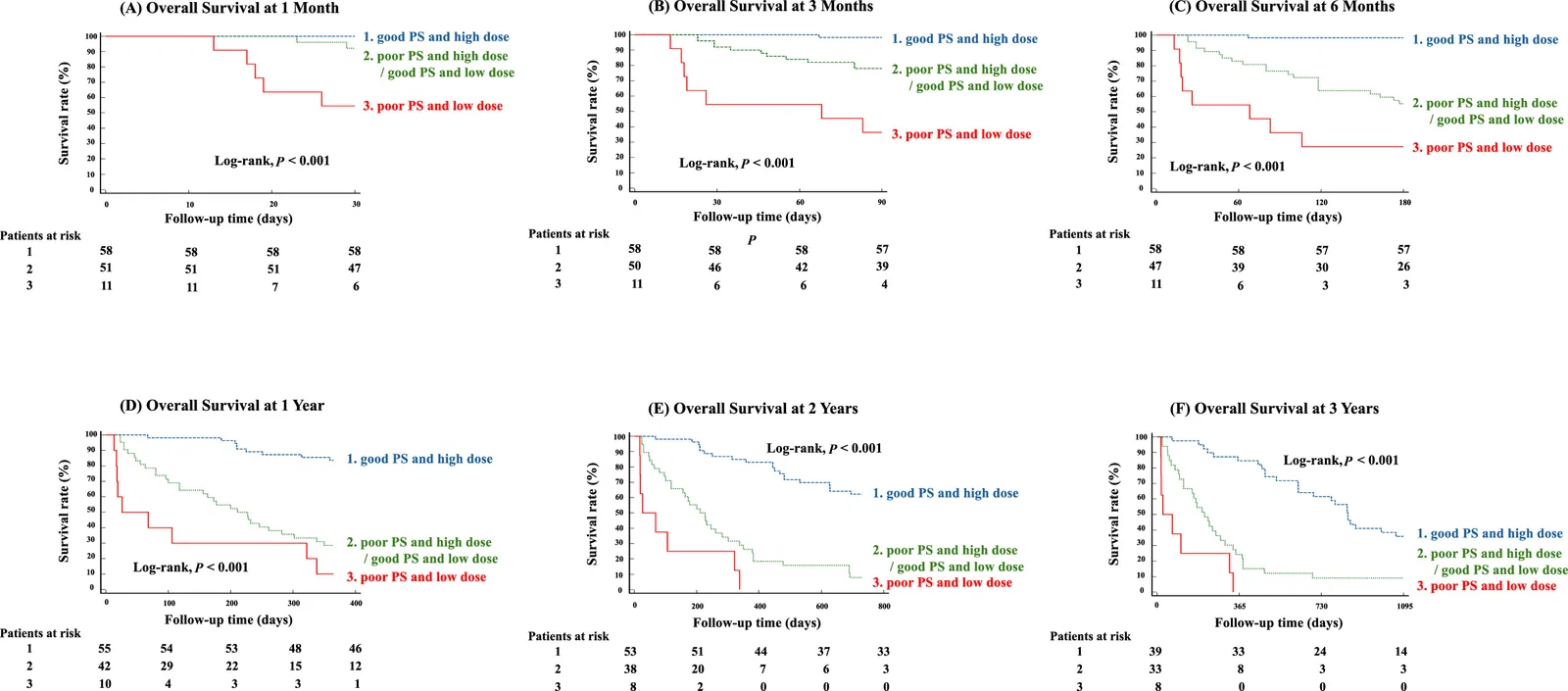
Kaplan–Meier curve analysis combining performance status (PS) and radiation dose in patients with low-risk primary tumor type. Kaplan–Meier curves showing overall survival in a subset of patients with low-risk primary tumor types, stratified by combinations of Eastern Cooperative Oncology Group Performance Status and prescribed radiation dose. Patients were categorized into 3 groups based on these 2 factors: group 1 included those with a good PS (<2) and a high prescribed dose (≥39 Gy in a biologically effective dose with α/β = 10); group 2 included patients with either a poor PS (≥2) and a high dose or a good PS and a low dose (<39 Gy in a biologically effective dose with α/β = 10); and group 3 included those with both a poor PS and a low dose. Survival was analyzed at (A) 1 month, (B) 3 months, (C) 6 months, (D) 1 year, (E) 2 years, and (F) 3 years posttreatment, with all comparisons showing statistically significant differences (log-rank *P* < .001).

**Figure 4:**
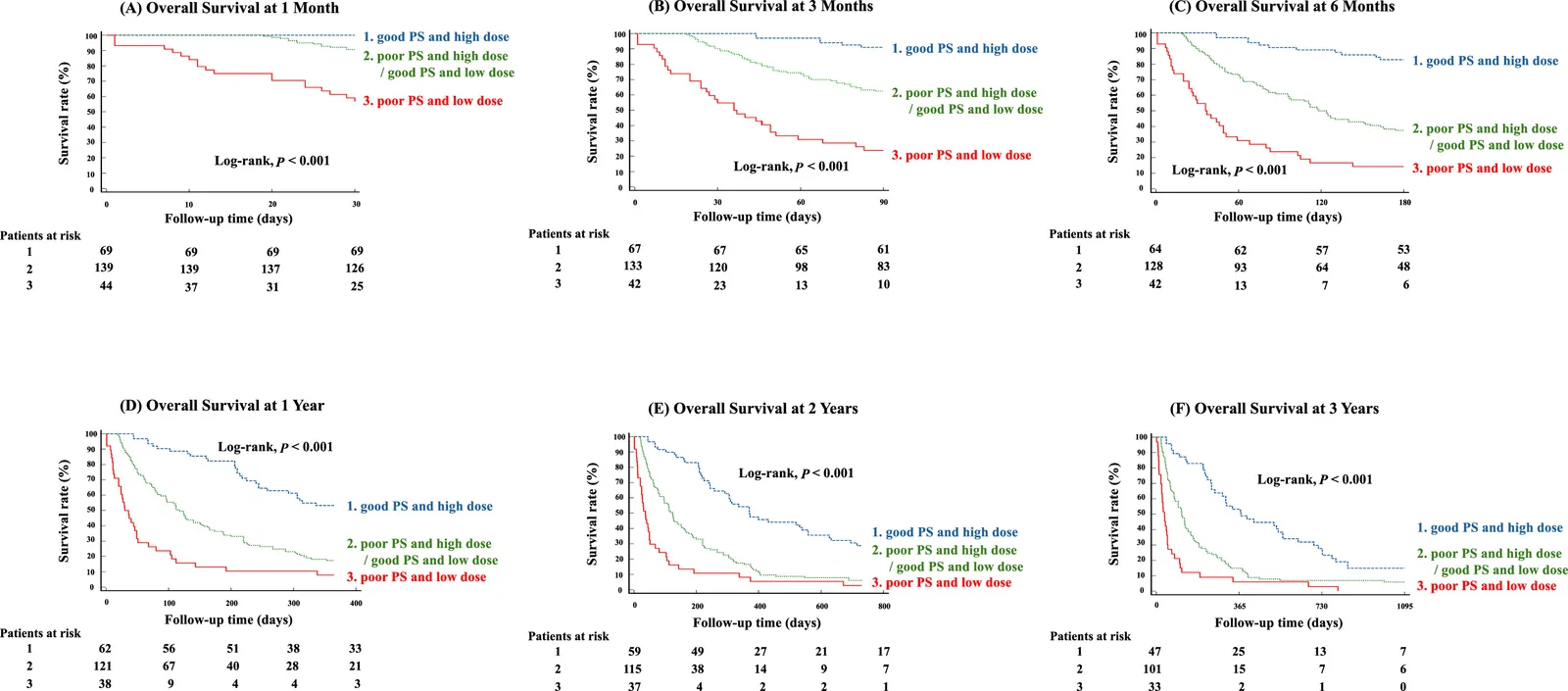
Kaplan–Meier curves combining performance status (PS) and radiation dose in patients with both bone and other-site metastases. Kaplan–Meier curves showing overall survival in a subset of patients with both bone and other-site metastases, stratified by combinations of Eastern Cooperative Oncology Group Performance Status and prescribed radiation dose. Patients were categorized into 3 groups based on these 2 factors: group 1 included those with a good PS (<2) and a high prescribed dose (≥39 Gy in a biologically effective dose with α/β = 10); group 2 included patients with either a poor PS (≥2) and a high dose or a good PS and a low dose (<39 Gy in a biologically effective dose with α/β = 10); and group 3 included those with both a poor PS and a low dose. Survival was analyzed at (A) 1 month, (B) 3 months, (C) 6 months, (D) 1 year, (E) 2 years, and (F) 3 years posttreatment, with all comparisons showing statistically significant differences (log-rank *P* < .001).

**Figure 5:**
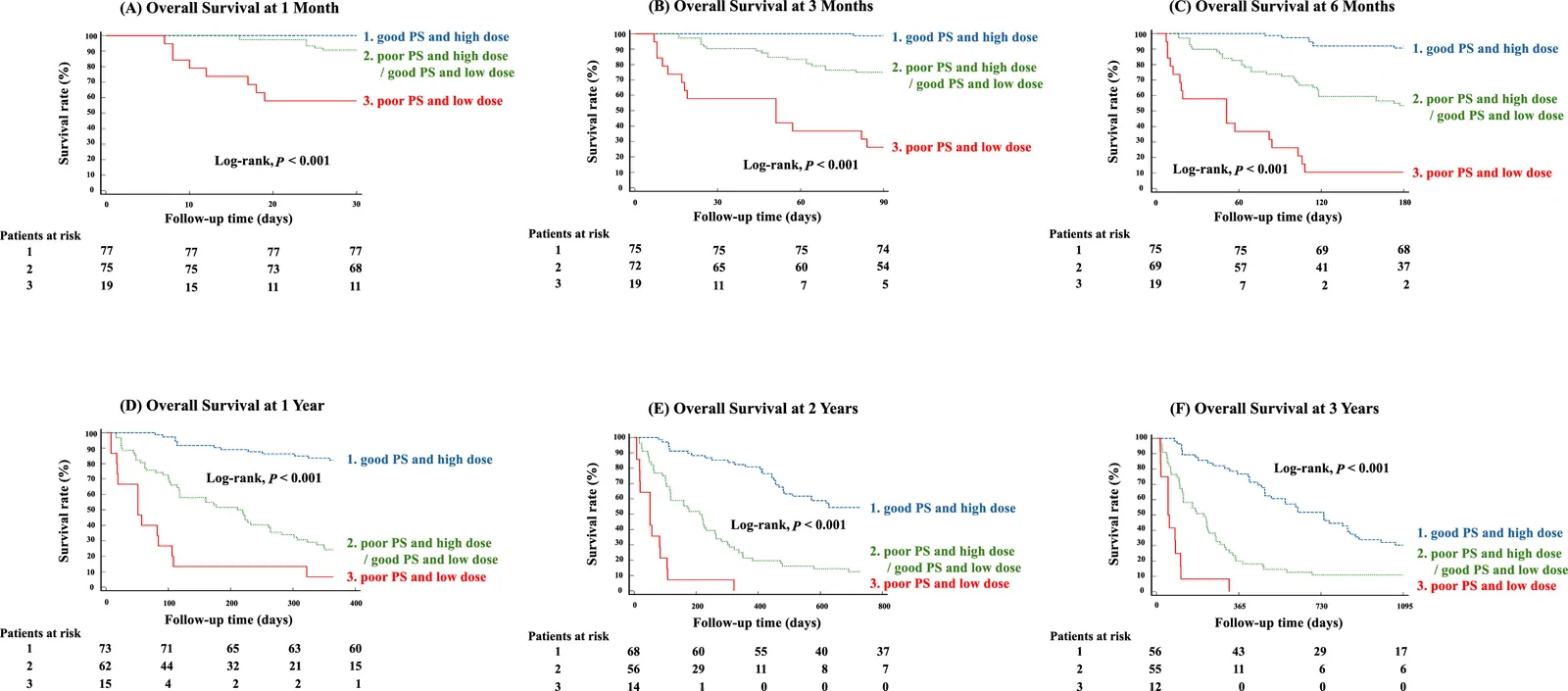
Kaplan–Meier curves combining performance status (PS) and radiation dose in patients with bone-only metastases. Kaplan–Meier curves showing overall survival in a subset of patients with bone-only metastases, stratified by combinations of Eastern Cooperative Oncology Group Performance Status and prescribed radiation dose. Patients were categorized into 3 groups based on these 2 factors: group 1 included those with a good PS (<2) and a high prescribed dose (≥39 Gy in a biologically effective dose with α/β = 10); group 2 included patients with either a poor PS (≥2) and a high dose or a good PS and a low dose (<39 Gy in a biologically effective dose with α/β = 10); and group 3 included those with both a poor PS and a low dose. Survival was analyzed at (A) 1 month, (B) 3 months, (C) 6 months, (D) 1 year, (E) 2 years, and (F) 3 years posttreatment, with all comparisons showing statistically significant differences (log-rank *P* < .001).

To confirm that our prognostic stratification was not driven solely by imminent mortality, we performed a sensitivity analysis excluding patients who died within 30 days of RT initiation. As shown in Fig. E3, significant survival stratification across the 3 groups persisted even after removing early deaths (log-rank *P* < .001).

To further investigate the dose-survival relationship, we performed a granular analysis, stratifying patients into 3 distinct groups based on BED10: <39 Gy, 39 Gy, and >39 Gy. As shown in Fig. E4, significant survival differences were observed across the 3 cohorts (log-rank *P* < .001). Patients receiving escalated doses (>39 Gy) had the most favorable survival outcomes, while those receiving <39 Gy had the poorest prognosis.

## Discussion

This study evaluated the combined prognostic value of PS and prescribed dose for OS in patients with bone metastases treated with palliative RT. The main findings can be summarized as follows: (1) the combination of PS and prescribed dose enabled more refined risk stratification for predicting both short-term and long-term OS; (2) the robustness of this combination’s prognostic significance was shown across key clinical subgroups, regardless of primary tumor risk classification or the number of distant metastatic sites; and (3) incorporating prescribed dose into PS yielded significant incremental predictive value for OS in these patients. Importantly, the observed dose-survival association should not be interpreted as evidence that a higher palliative RT dose directly prolongs survival. Because the prescribed dose was selected in routine clinical practice, it may function as a treatment-related prognostic marker that partly reflects clinical factors considered by radiation oncologists during regimen selection but not fully captured in our database. These factors may include metastatic burden, available systemic therapy options, symptom context, feasibility of repeated visits, and the anticipated need for durable local palliation. Thus, improvement in AUC after adding prescribed dose to PS likely reflects, at least in part, prognostic information embedded in real-world treatment selection.

PS is a fundamental clinical parameter in oncology, widely used to inform therapeutic decision-making and prognostic assessment. Its important role in research is further underscored by its routine inclusion as an eligibility criterion in clinical trials.^13^ In palliative RT for bone metastases, substantial evidence shows a strong correlation between favorable PS and improved survival, making it a core component of many prognostic models.^14^, ^15^, ^16^^,^^24^, ^25^, ^26^, ^27^, ^28^ For instance, Chow et al^28^ developed and externally validated a prognostic model for patients with advanced cancer referred for palliative RT, demonstrating that PS was one of the strongest predictors of survival. Similarly, Katagiri et al’s^14^ scoring system identified PS as a foundational determinant of survival in patients with bone metastases. Building on this foundation, the present study extends the use of PS by evaluating the incremental prognostic value of prescribed radiation dose in patients with bone metastases. While PS remains a critical predictor of OS in patients receiving palliative RT, our receiver operating characteristic (ROC) curve analysis demonstrated that incorporating prescribed dose into PS provided significant incremental predictive value for OS. The combined PS and prescribed dose framework may help identify prognostic subgroups observed in routine palliative RT practice; however, it should not be interpreted as a stand-alone decision-making tool for selecting higher-dose regimens because its prognostic value may reflect clinical factors that influence regimen selection.

The optimal fractionation schedule for palliative RT in bone metastases has been a subject of longstanding debate. Current international guidelines reflect this situation, endorsing regimens ranging from a single fraction of 8 Gy to multifraction schedules such as 20 Gy in 5 fractions or 30 Gy in 10 fractions.^20^ Nevertheless, several retrospective observational studies have reported an association between higher radiation doses and longer survival.^17^, ^18^, ^19^ Chou et al^17^ conducted a large propensity score-matched analysis of 1795 patients with bone metastases from solid tumors and reported that dose-escalated RT (equivalent dose in 2 Gy fractions >32.5 Gy) was associated with better survival outcomes than standard-dose treatment (30 Gy in 10 fractions; equivalent dose in 2 Gy fractions = 32.5 Gy).^17^ This favorable prognostic association was most pronounced among patients with nonlung cancer, the absence of multiple metastases, no presenting symptoms, and a favorable prognosis. Similarly, Kao et al^18^ reported that, among patients with various distant metastases receiving palliative RT, higher radiation dose intensity was associated with better survival outcomes. However, these findings should be interpreted cautiously because patients with a more favorable expected prognosis may have been preferentially selected for longer-course or higher-dose regimens. Therefore, the observed dose-survival relationship may at least partly reflect selection bias and residual confounding rather than a direct survival benefit of higher-dose palliative RT. Despite these findings, the prognostic role of a radiation regimen remains unclear. In contrast to PS, a near-universal prognostic tool in this setting, a specific fractionation schedule has not consistently emerged as an independent predictor of survival in previous studies and is absent from several prognostic models.^29^^,^^30^

To elucidate the dose-survival relationship in this study, we used a BED10 cutoff of 39 Gy, corresponding to the standard palliative regimen of 30 Gy in 10 fractions widely adopted during the study period. This threshold serves as a logical delineation between standard and dose-escalated regimens, a categorization supported by previous literature.^31^^,^^32^ While the primary clinical rationale for escalating doses beyond this standard is to improve local control, our granular analysis stratifying patients into 3 groups (<39 Gy, 39 Gy, and >39 Gy) demonstrated that patients receiving higher doses had superior survival outcomes, reinforcing the prognostic significance of radiation dose intensity beyond the standard regimen.

It is important to note the temporal limitations of our model’s predictive accuracy. While incorporating the prescribed dose into PS significantly improved the AUC for OS at 1, 3, and 6 months, as well as at 1 and 2 years, this incremental prognostic value did not reach statistical significance at 3 years. This finding implies that the prognostic value of the palliative radiation dose, primarily mediated through local control and symptom stabilization, is most potent in the short- to intermediate-term. For long-term survivors, other determinants, such as the emergence of new distant metastases and response to subsequent systemic therapies, may become the dominant drivers of survival, diluting the impact of the initial RT regimen.

### Clinical implications

As noted above, while previous studies have demonstrated the individual prognostic value of PS and prescribed dose for OS in patients receiving palliative RT, this study’s central contribution is showing that combining these 2 factors enabled more refined risk stratification for both short- and long-term survival. This integrated approach also identified a subgroup of patients with intermediate prognosis, whose survival outcomes fell between those of the favorable and poor prognosis groups. This intermediate-risk cohort challenges the practice of determining radiation dose based solely on PS. In these patients, a higher prescribed dose was associated with better survival outcomes, suggesting its potential utility as a prognostic marker. Pain response at the irradiated site was evaluated as a local symptomatic outcome according to international consensus criteria defined in Tables E2 and E3. As shown in Table E4, overall pain response rates, defined as a complete or partial response, were 49.7% at 1 month, 48.5% at 3 months, and 48.9% at 6 months after RT. When comparing overall response with pain progression, no statistically significant differences were observed across the 3 prognostic groups at any time point. These findings suggest that the observed survival stratification was not accompanied by clear between-group differences in local pain response and pain progression. Importantly, our supplementary analysis reinforces the value of this approach in the intermediate-risk cohort: compared with the poor-prognosis cohort, these patients had significantly lower rates of functional deterioration. Although higher doses were associated with a modest increase in acute adverse events, they were exclusively mild (grade 1-2) with no severe toxicities observed, suggesting a favorable risk-benefit profile. Nevertheless, it should be noted that the survival of the intermediate-risk cohort remained inferior to that of the favorable prognosis cohort. Therefore, clinicians should carefully weigh the observed association between a higher prescribed dose and longer survival against the disadvantages of longer-course RT in practice. Nevertheless, this combined assessment framework enables more flexible, individualized therapeutic decisions, allowing clinicians to tailor fractionation schedules to a patient’s specific clinical context and prognostic profile. Importantly, the consistency of these findings across subgroups stratified by primary tumor risk and the extent of metastatic disease suggests the broad applicability of this integrated prognostic approach across diverse clinical scenarios.

### Limitations

This study has some limitations. First, its retrospective, single-institution design may limit the external validity of our findings. Second, the study population was heterogeneous in systemic treatment status, including patients who received various systemic agents or RT alone, which could have acted as potential confounders. Furthermore, this study did not account for the specific type, duration, or sequencing of systemic therapies, such as immune checkpoint inhibitors and molecular targeted agents. Given the profound impact of these treatments on survival, this unmeasured heterogeneity is a significant confounder that limits our ability to conclusively attribute the observed prognostic outcomes solely to PS and radiation dose. Therefore, given our retrospective design and these unmeasured confounders, causality cannot be established, and the prescribed radiation dose should be interpreted primarily as a prognostic marker rather than a causal, survival-modifying treatment. Third, while primary tumors were categorized into 2 risk groups based on previous literature,^14^^,^^16^^,^^21^^,^^22^ other prognostic classification approaches have also been proposed.^15^^,^^28^^,^^30^ Therefore, prospective, multicenter studies are warranted to validate these findings in a more controlled environment and confirm the broad applicability of our approach. Fourth, the choice of fractionation regimen was likely influenced by clinical assessment of life expectancy not fully captured by PS, such as the exhaustion of systemic treatment options. Consequently, potential confounding by indication remains a limitation, as patients with a poorer prognosis might have been preferentially selected for lower BEDs regardless of PS. Fifth, detailed molecular data, including driver mutation status in lung cancer and molecular subtypes in breast cancer, were not uniformly available. This heterogeneity in tumor biology may have acted as an unmeasured confounder impacting OS. Sixth, BED calculation relied on a uniform α/β ratio of 10 Gy for all tumor types. This assumption may not accurately reflect the radiobiological characteristics of certain histologies, such as prostate cancer, which typically exhibit lower α/β ratios, potentially leading to inaccurate estimates of the biological dose for these subgroups. Seventh, the incremental predictive value of adding prescribed dose to PS diminished over time and was not statistically significant at 3 years. This indicates that the combined model is less effective for long-term prognostication, where systemic disease factors likely outweigh the prognostic significance of the initial palliative RT dose. Eighth, lesion-level metastatic counts and longitudinal progression status were not consistently available. Therefore, patients with true oligometastatic or oligoprogressive disease could not be rigorously identified or analyzed separately. Although we performed an exploratory supplementary analysis stratified by the number of nonbone metastatic organ-site categories (≤1 vs ≥2), this should be interpreted as a surrogate measure of metastatic burden rather than a formal classification of oligometastatic or oligoprogressive disease. Ninth, radiographic local control at the irradiated site was not consistently available in this retrospective cohort. Therefore, we evaluated pain response at the irradiated site as a local symptomatic outcome; however, this should not be interpreted as a direct measure of radiographic tumor control.

## Conclusions

Combining PS and prescribed dose enabled more refined risk stratification for predicting both short- and long-term OS in patients with bone metastases undergoing palliative RT, regardless of primary tumor risk classification or the number of distant metastases. Moreover, incorporating prescribed dose into PS yielded significant incremental predictive value for OS in this setting compared with PS alone.

## Disclosures

None.

## Appendix Supplementary materials

Supplementary material_clean Image, application 1

## Footnotes

Supplementary material associated with this article can be found in the online version at doi:10.1016/j.adro.2026.102170.
