# Supplementary material for: Incremental Value of Radiation Dose for Prognostic Stratification Beyond Performance Status in Palliative Radiation Therapy for Bone Metastases
Source: Adv Radiat Oncol. 2026 Oct 4;11(12):102170. doi: 10.1016/j.adro.2026.102170 (PMC13659670; doi:10.1016/j.adro.2026.102170)
Supplement: Supplementary file 1 [file mmc1.docx]

**Figure E1. Forest plot of hazard ratios for 1-year overall survival based on prescribed dose across clinical subgroups**

The figure shows a forest plot that demonstrates results of subgroup analyses for 1-year OS in patients stratified by key clinical variables to assess the robustness of the prescribed dose as a prognostic factor. The forest plot illustrates the hazard ratios associated with a lower prescribed dose (<39 Gy in BED10) within these subgroups, which includes age, sex, number of distant metastases, and the status of specific metastatic sites. A consistent trend was observed across all evaluated subgroups: a lower prescribed dose was associated with poorer survival.

BED10, biologically effective dose with α/β = 10; OS, overall survival.

**Figure E2. Kaplan-Meier Estimates of 1-Year Overall Survival by Prescribed Dose in Patients Stratified by the Number of Non-Bone Metastatic Site Categories**

Kaplan-Meier curves of 1-year overall survival stratified by prescribed dose are shown for patients with ≤1 non-bone metastatic site category (A) and ≥2 non-bone metastatic site categories (B). In both subgroups, patients receiving a lower prescribed dose (<39 Gy in BED10) had significantly poorer survival than those receiving a higher prescribed dose (≥39 Gy in BED10) (both log-rank p < 0.001).

**Figure E3. Kaplan-Meier estimates of overall survival excluding early mortality (deaths within 30 days).**

Survival curves are stratified by the combination of baseline performance status and prescribed radiation dose, excluding patients who died within 30 days of treatment initiation. Group 1 (blue line) represents patients with good PS and high dose; Group 2 (green line) represents patients with intermediate prognosis (poor PS/high dose or good PS/low dose); and Group 3 (red line) represents patients with poor PS and low dose. The log-rank test indicates a statistically significant difference among the three groups (p < 0.001).

PS = Performance Status

**Figure E4. Kaplan-Meier Estimates of Overall Survival Stratified by Three Prescribed Dose Levels (<39 Gy, 39 Gy, and >39 Gy)**

Survival curves are stratified solely by prescribed radiation dose (BED10). The blue line represents patients receiving escalated doses (>39 Gy); the green line represents patients receiving the standard dose (39 Gy); and the red line represents patients receiving lower doses (<39 Gy). The log-rank test indicates a statistically significant difference among the three groups (p < 0.001), demonstrating that higher radiation doses are associated with longer survival durations.

**Table E1. Distribution of patients according to the number of non-bone metastatic site categories**

| Number of non-bone metastatic site categories | No. of patients (%)  (n = 423) |
| --- | --- |
| 0  1  2  3  4  5  6 | 159 (37.6)  117 (27.7)  70 (16.5)  35 (8.3)  30 (7.1)  9 (2.1)  3 (0.7) |

Because all patients in this cohort had bone metastases, only non-bone metastatic site categories were counted. Non-bone metastatic site categories included brain, lung, liver, adrenal gland, non-regional lymph nodes, pleural dissemination, peritoneal dissemination, and other sites. Other sites included subcutaneous, pancreatic, renal, chest wall, muscle, and other less frequent metastatic sites. This count represents the number of involved metastatic site categories, not the total number of individual metastatic lesions.

**Table E2. Definition of Analgesic scoring system**

| Term | Definition |
| --- | --- |
| Score 0 | Analgesic not required. |
| Score 1 | Nonopioid analgesic irregularly required. |
| Score 2 | Nonopioid analgesics regularly required. |
| Score 3 | Oral or parenteral opioids occasionally required. |
| Score 4 | Oral or parenteral opioids regularly required. |

Reference

#1.Duraisamy, Ina Shaliny, Marniza Saad, and Adlinda Alip. Single vs multiple fraction palliative radiotherapy for uncomplicated painful bone metastases treated at University of Malaya Medical Centre: A single institutional Malaysian experience. Aging Medicine 2018;1(2): 133-140.

**Table E3. Definitions of pain response categories based on International Consensus Endpoints**

| Term | Definition |
| --- | --- |
| Complete response | A pain score of 0 at the treated site with no increase in analgesic intake. |
| Partial response | A reduction in pain score by at least 2 points on the numerical rating scale (NRS) without an increase in analgesic score*, or a reduction in analgesic score or analgesic dose by ≥ 25% from baseline without an increase in pain. |
| Pain progression | An increase in pain score by at least 2 points above baseline at the treated site with stable analgesic use, or an increase in analgesic score or analgesic dose by ≥ 25% from baseline without a decrease in pain. |
| Indeterminate response | Any response not classified as complete response, partial response, or pain progression. |

Pain response and analgesic scoring were evaluated based on the International Consensus Endpoints, using the numerical rating scale (NRS) for pain assessment.

*The analgesic score was defined according to the analgesic scoring system described in Table E2.

References

#1. Duraisamy, Ina Shaliny, Marniza Saad, and Adlinda Alip. Single vs multiple fraction palliative radiotherapy for uncomplicated painful bone metastases treated at University of Malaya Medical Centre: A single institutional Malaysian experience. Aging Medicine 2018;1(2): 133-140.

#2. Chow E, Hoskin P, Mitera G, Zeng L, Lutz S, Roos D, et al. Update of the international consensus on palliative radiotherapy endpoints for future clinical trials in bone metastases. Int J Radiat Oncol Biol Phys. 2012;82(5):1730-7

**Table E4. Pain response rates according to the combination of baseline performance status and prescribed radiation dose**

| International consensus endpoint | All patients | Group 1 | Group 2 | Group 3 | P value** |
| --- | --- | --- | --- | --- | --- |
| 1 month*  Overall response  CR  PR  Pain progression  Indeterminate response | (n = 326)  162 (49.7)  73 (22.4)  89 (27.3)  74 (22.7)  90 (27.6) | (n = 138)  61 (44.2)  36 (26.1)  25 (18.1)  29 (21.0)  48 (34.8) | (n = 158)  85 (53.8)  31 (19.6)  54 (34.2)  38 (24.1)  35 (22.2) | (n = 30)  16 (53.3)  6 (20.0)  10 (33.3)  7 (23.3)  7 (23.3) | 0.974 |
| 3 months*  Overall response  CR  PR  Pain progression  Indeterminate response | (n = 239)  116 (48.5)  58 (24.3)  58 (24.3)  56 (23.4)  67 (28.0) | (n = 123)  61 (49.6)  35 (28.5)  26 (21.1)  27 (22.0)  35 (28.5) | (n = 104)  48 (46.2)  20 (19.2)  28 (26.9)  27 (26.0)  29 (27.9) | (n = 12)  7 (58.3)  3 (25.0)  4 (33.3)  2 (16.7)  3 (25.0) | 0.612 |
| 6 months*  Overall response  CR  PR  Pain progression  Indeterminate response | (n = 188)  92 (48.9)  45 (23.9)  47 (25.0)  44 (23.4)  52 (27.7) | (n = 113)  56 (49.6)  33 (29.2)  23 (20.4)  22 (19.5)  35 (31.0) | (n = 69)  32 (46.4)  11 (15.9)  21 (30.4)  22 (31.9)  15 (21.7) | (n = 6)  4 (66.7)  1 (16.7)  3 (50.0)  0 (0.0)  2 (33.3) | 0.119 |

Patients were categorized into three groups based on Eastern Cooperative Oncology Group performance status (ECOG PS) and prescribed radiation dose. Group 1 included patients with good PS (< 2) and a high prescribed dose (≥ 39 Gy in biologically effective dose with α/β = 10 [BED10]). Group 2 included patients with either poor PS (≥ 2) and a high dose, or good PS and a low dose (< 39 Gy in BED10). Group 3 included patients with both poor PS and a low dose.

Values are n (%). BED10, biologically effective dose with α/β = 10; CR, complete response; PR, partial response; Overall response was defined as CR or PR; Indeterminate response was defined as any response not classified as CR, PR, or pain progression.

*Treatment responses were evaluated at 1, 3, and 6 months after radiotherapy.

**P value is the result of Chi-square test for “Overall response” vs. “Pain progression”.

**Table E5. Summary of acute adverse events related to palliative radiotherapy stratified by prognostic groups (Group 1-3)**

| Adverse events | All patients  (n = 423) | Group 1  (n = 146) | Group 2  (n = 214) | Group 3  (n = 63) | P value |
| --- | --- | --- | --- | --- | --- |
| CTCAE grade  Grade 0  Grade 1  Grade 2  Grade ≥ 3 | 335 (79.2)  63 (14.9)  25 (5.9)  0 (0.0) | 106 (72.6)  30 (20.5)  10 (6.8)  0 (0.0) | 168 (78.5)  32 (15.0)  14 (6.5)  0 (0.0) | 61 (96.8)  1 (1.6)  1 (1.6)  0 (0.0) | 0.003 |
| Pain flare | 2 (0.5) | 1 (0.7) | 1 (0.5) | 0 (0.0) | N.A. |

Patients were categorized into three groups based on Eastern Cooperative Oncology Group performance status (ECOG PS) and prescribed radiation dose. Group 1 included patients with good PS (< 2) and a high prescribed dose (≥ 39 Gy in biologically effective dose with α/β = 10 [BED10]). Group 2 included patients with either poor PS (≥ 2) and a high dose, or good PS and a low dose (< 39 Gy in BED10). Group 3 included patients with both poor PS and a low dose.

Values are n (%). CTCAE, Common Terminology Criteria for Adverse Events; N.A., not applicable.

Acute adverse events which occur during radiotherapy or within 90 days after treatment completion were graded according to the Common Terminology Criteria for Adverse Events (CTCAE). Only the maximum grade per patient was recorded.

**Table E6. Detailed breakdown of acute adverse events related to palliative radiotherapy by toxicity types and prognostic groups (Group 1-3)**

| Adverse events | All patients  (n = 423) | Group 1  (n = 146) | Group 2  (n = 214) | Group 3  (n = 63) |
| --- | --- | --- | --- | --- |
| Gastrointestinal  Nausea/Decreased appetite  Grade 1  Grade 2  Diarrhea  Grade 1  Grade 2  Proctitis  Grade 1  Grade 2  Mucositis  (oral, pharyngeal,  laryngeal, esophageal)  Grade 1  Grade 2  Genitourinary  Cystitis/urethritis  Grade 1  Grade 2  Dermatitis  Grade 1  Grade 2  Others  Fatigue  Grade 1  Grade 2  Hematologic toxicity  Grade 1  Grade 2  Tympanitis  Grade 1  Grade 2 | 5 (1.2)  6 (1.4)  6 (1.4)  6 (1.4)  0 (0.0)  1 (0.2)  32 (7.6)  6 (1.4)  1 (0.2)  2 (0.5)  26 (6.1)  6 (1.4)  1 (0.2)  0 (0.0)  0 (0.0)  2 (0.5)  1 (0.2)  0 (0.0) | 2 (1.4)  2 (1.4)  4 (2.7)  3 (2.1)  0 (0.0)  1 (0.7)  13 (8.9)  1 (0.7)  1 (0.7)  2 (1.4)  11 (7.5)  2 (1.4)  0 (0.0)  0 (0.0)  0 (0.0)  1 (0.7)  1 (0.7)  0 (0.0) | 3 (1.4)  3 (1.4)  2 (0.9)  3 (1.4)  0 (0.0)  0 (0.0)  19 (8.9)  5 (2.3)  0 (0.0)  0 (0.0)  14 (6.5)  4 (1.9)  1 (0.5)  0 (0.0)  0 (0.0)  1 (0.5)  0 (0.0)  0 (0.0) | 0 (0.0)  1 (1.6)  0 (0.0)  0 (0.0)  0 (0.0)  0 (0.0)  0 (0.0)  0 (0.0)  0 (0.0)  0 (0.0)  1 (1.6)  0 (0.0)  0 (0.0)  0 (0.0)  0 (0.0)  0 (0.0)  0 (0.0)  0 (0.0) |

Patients were categorized into three groups based on Eastern Cooperative Oncology Group performance status (ECOG PS) and prescribed radiation dose. Group 1 included patients with good PS (< 2) and a high prescribed dose (≥ 39 Gy in biologically effective dose with α/β = 10 [BED10]). Group 2 included patients with either poor PS (≥ 2) and a high dose, or good PS and a low dose (< 39 Gy in BED10). Group 3 included patients with both poor PS and a low dose.

Values are n (%). CTCAE, Common Terminology Criteria for Adverse Events.

Acute adverse events were graded according to the Common Terminology Criteria for Adverse Events (CTCAE), and no grade ≥ 3 acute adverse events were observed.

Nausea/decreased appetite includes patients with nausea and/or decreased appetite.

**Table E7. Changes in ECOG performance status at one-month post-radiotherapy**

| PS change status | All patients  (n = 326) | Group 1  (n = 138) | Group 2  (n = 158) | Group 3  (n = 30) | P value |
| --- | --- | --- | --- | --- | --- |
| Improved  (PS improved by ≥ 1) | 9 (2.8) | 0 (0.0) | 7 (4.4) | 2 (6.7) | 0.004 |
| Stable  (PS unchanged) | 287 (88.0) | 122 (88.4) | 143 (90.5) | 22 (73.3) |  |
| Worsened  (PS worsened by ≥ 1) | 30 (9.2) | 16 (11.6) | 8 (5.1) | 6 (20.0) |  |

Patients were categorized into three groups based on Eastern Cooperative Oncology Group performance status (ECOG PS) and prescribed radiation dose. Group 1 included patients with good PS (< 2) and a high prescribed dose (≥ 39 Gy in biologically effective dose with α/β = 10 [BED10]). Group 2 included patients with either poor PS (≥ 2) and a high dose, or good PS and a low dose (< 39 Gy in BED10). Group 3 included patients with both poor PS and a low dose.

Values are n (%). ECOG PS, Eastern Cooperative Oncology Group Performance Status; PS, performance status.

*Changes in ECOG PS from baseline were evaluated at 1 month after radiotherapy.

**Table E8. Baseline distribution of ECOG performance status within each prognostic group**

| ECOG PS | All patients  (n = 423) | Group 1  (n = 146) | Group 2  (n = 214) | Group 3  (n = 63) | P value |
| --- | --- | --- | --- | --- | --- |
| PS 0–1 | 152 (35.9) | 146 (100.0) | 6 (2.8) | 0 (0.0) | < 0.001*  0.123** |
| PS 2 | 104 (24.6) |  | 85 (39.7) | 19 (30.2) |  |
| PS 3–4 | 167 (39.5) |  | 123 (57.5) | 44 (69.8) |  |

Patients were categorized into three groups based on Eastern Cooperative Oncology Group performance status (ECOG PS) and prescribed radiation dose. Group 1 included patients with good PS (< 2) and a high prescribed dose (≥ 39 Gy in biologically effective dose with α/β = 10 [BED10]). Group 2 included patients with either poor PS (≥ 2) and a high dose, or good PS and a low dose (< 39 Gy in BED10). Group 3 included patients with both poor PS and a low dose.

Values are n (%). ECOG PS, Eastern Cooperative Oncology Group Performance Status; PS, performance status.

*This P value is the result of Chi-square test for all Groups.

**This P value is the result of Chi-square test for Group 2 vs. Group 3.
